# A retrospective observational study of osteoporosis management after a fragility fracture in primary care

**DOI:** 10.1007/s11657-022-01110-z

**Published:** 2022-05-06

**Authors:** Alan Bell, David L. Kendler, Aliya A. Khan, Marla Shapiro C.M., Anne Morisset, Jean-Pierre Leung, Maureen Reiner, Stephen M. Colgan, Lubomira Slatkovska, Millicent Packalen

**Affiliations:** 1 Department of Family and Community Medicine, University of Toronto, Toronto, ON Canada; 2 Department of Medicine, Division of Endocrinology, University of British Columbia, Vancouver, BC Canada; 3 Department of Medicine, Divisions of Endocrinology and Metabolism and Geriatrics, McMaster University, Hamilton, ON Canada; 4 Department of Medicine, Division of Internal Medicine, Sherbrooke University, Sherbrooke, QC Canada; 5 Department of Family Medicine, University of Calgary, Calgary, AB Canada; 6 Amgen Inc, Thousand Oaks, CA USA; 7 Amgen Canada Inc, Mississauga, ON Canada

**Keywords:** Osteoporosis, Fracture, Secondary prevention, Primary care, Real-world data

## Abstract

**Summary:**

In many countries, osteoporosis is predominantly managed by primary care physicians; however, management after a fragility fracture has not been widely investigated. We describe osteoporosis care gaps in a real-world patient cohort. Our findings help inform initiatives to identify and overcome obstacles to effective management of patients after fragility fracture.

**Purpose:**

A fragility fracture is a major risk factor for subsequent fracture in adults aged ≥ 50 years. This retrospective observational study aimed to characterize post-fracture management in Canadian primary care.

**Methods:**

A total of 778 patients with an index fragility fracture (low-trauma, excluding small bones) occurring between 2014 and 2016 were identified from medical records at 76 primary care centers in Canada, with follow-up until January 2018.

**Results:**

Of 778 patients (80.5% female, median age [IQR] 73 [64–80]), 215 were on osteoporosis treatment and 269 had osteoporosis diagnosis recorded prior to their index fracture. The median follow-up was 363 (IQR 91–808) days. Of patients not on osteoporosis treatment at their index fracture, 60.2% (*n* = 339/563) remained untreated after their index fracture and 62.2% (*n* = 23/37) continued untreated after their subsequent fracture. After their index fracture, fracture risk assessment (FRAX or CAROC) was not performed in 83.2% (*n* = 647/778) of patients, and 59.9% (*n* = 466/778) of patients did not receive bone mineral density testing. Of patients without osteoporosis diagnosis recorded prior to their index date, 61.3% (*n* = 300/489) remained undiagnosed after their index fracture. At least one subsequent fracture occurred in 11.5% (*n* = 86/778) of patients.

**Conclusion:**

In the primary care setting, fragility fracture infrequently resulted in osteoporosis treatment or fracture risk assessment, even after multiple fragility fractures. These results suggest a fragility fracture is not recognized as a major risk factor for subsequent fracture and its occurrence does not prompt primary care physicians to intervene. These data urge initiatives to identify and overcome obstacles to primary care physicians’ effective management of patients after fragility fractures.

**Supplementary Information:**

The online version contains supplementary material available at 10.1007/s11657-022-01110-z.

## Background

Fragility fracture warrants a clinical diagnosis of osteoporosis [1, 2], yet osteoporosis remains under-diagnosed and under-treated in the secondary fracture prevention setting worldwide [3, 4]. With global incidence, morbidity, mortality, and costs of fragility fractures expected to increase with the aging population [5, 6], osteoporosis is currently recognized as a major public health concern [5, 7–9]. The annual incidence of fragility fractures in Canada and the USA is estimated to be higher than that for myocardial infarction, stroke, and breast cancer combined [10–12], while the excess 1-year mortality associated with a fragility fracture is estimated to be comparable to that for myocardial infarction [13]. Direct healthcare costs attributed to fragility fracture within the first year post-fracture are estimated at $1.9 billion in Canada [14].

In many countries, including Canada, osteoporosis is predominantly managed by primary care physicians (PCP) [15, 16], with more complex cases being referred to specialists. PCP management after an incident fragility fracture has not been widely investigated, with most existing data collected prior to shifts in recent guidelines for the clinical management of osteoporosis [17–20]. After the availability of 10-year fracture risk assessment tools in 2008 (i.e., the Fracture Risk Assessment Tool [FRAX] and the Canadian Association of Radiologists and Osteoporosis Canada [CAROC] tool), management of osteoporosis relied on both bone mineral density (BMD) and 10-year fracture risk, incorporating fracture history along with BMD, age, and other risk factors [21]. Furthermore, recent fragility fracture (≤ 2 years) is associated with a very high or imminent risk of subsequent fracture [1, 22]. For instance, median time to subsequent fragility fracture was recently estimated to be 555 (IQR 236–955) days following index fracture in Canadian adults aged > 65 years [23]. This imminent fracture risk is not captured in FRAX or CAROC, but should motivate more aggressive osteoporosis treatment in patients soon after their fragility fracture. Indeed, this treatment paradigm is reflected in recent international guidelines [1, 22, 24]. Specifically, recent North American guidelines recommend initiation of pharmacotherapy in post-menopausal women soon after an incident fragility fracture, and more potent pharmacotherapies (i.e., bone formation therapies, denosumab or zoledronic acid) are recommended first-line [1, 22]. Recent guidelines germane to our study also validate that a clinical diagnosis of osteoporosis based on a history of fragility fracture and independent of BMD should prompt treatment initiation [1, 2].

The primary objective of this real-world retrospective cohort study was to characterize osteoporosis treatment patterns in the primary care setting after incident fragility fracture. The secondary objectives were to describe fracture risk assessment, BMD assessment, and the establishment of osteoporosis diagnosis in patients with fragility fracture. For both objectives, the post-fracture care gap was characterized in the context of the 2010 Canadian osteoporosis guidelines, applicable to the surveillance period and geographic origin of this post-fragility fracture cohort [21].

## Methods

### Study design and setting

**Fig. 1:**
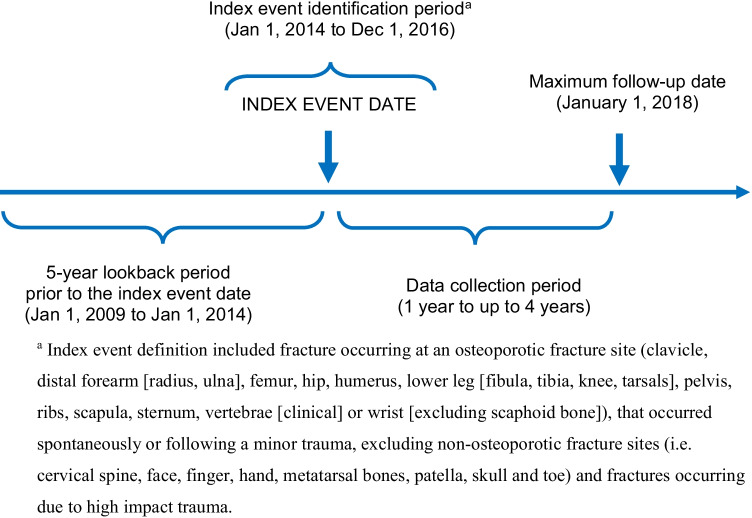
Study schema

We conducted a retrospective chart review study in primary care centers across Canada (Fig. 1). PCPs identified by the study sponsor and its research support agencies were recruited for this study. Those who expressed initial interest were evaluated through a site visit based on criteria related to their center’s research capabilities (e.g., clinic resources, number of potential patients, and physicians’ research experience). Financial compensation was provided to participating sites for each patient reviewed, and data were extracted from patient electronic or paper medical records. The study protocol was approved by the Advarra ethics review board (74 sites) or by site-specific institutional review board (2 sites).

### Study participants

**Fig. 2:**
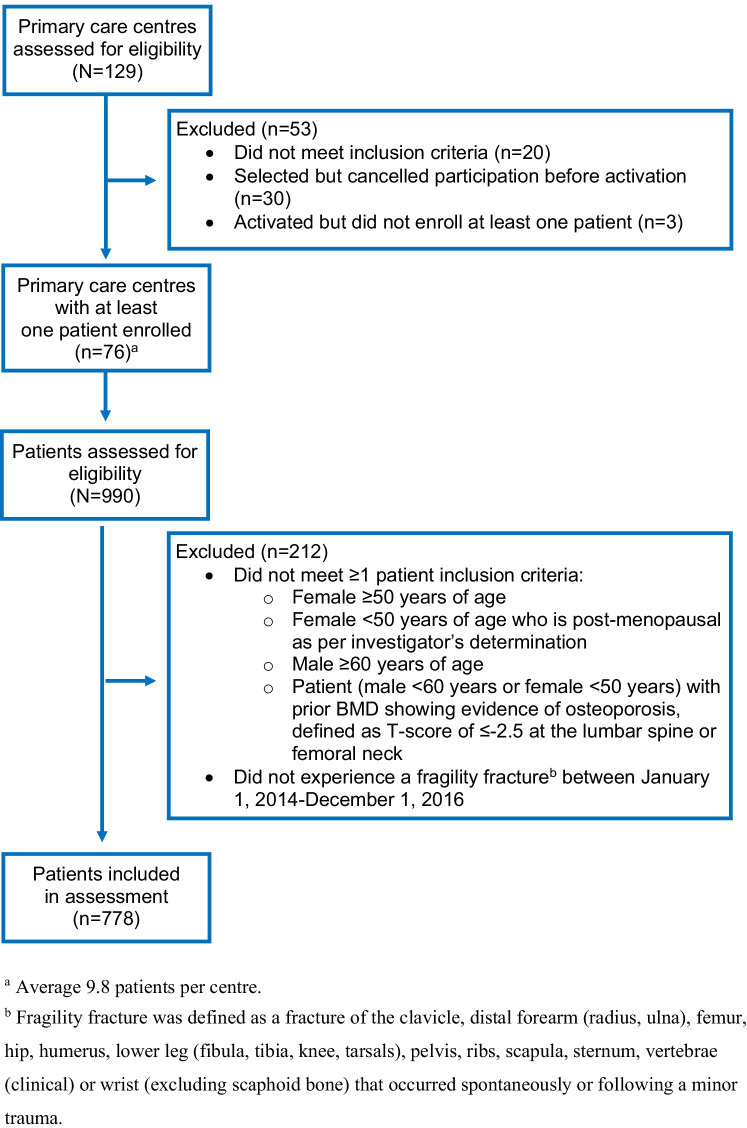
Flow diagram of sites and patients included in the index fracture cohort

Eligible patients (1) experienced an index fragility fracture between January 1, 2014 and December 1, 2016, defined as a fracture that occurred spontaneously or following minor trauma at the hip, radius, ulna, humerus, spine (symptomatic vertebral fracture), wrist (distal forearm), femur, lower leg (tibia, fibula, knee, tarsal bones), ribs, shoulder, arm, sternum, clavicle, or pelvis; and (2) at the time of their index fracture were (i) female ≥ 50 years, (ii) female < 50 years and post-menopausal (as per the investigator’s determination), (iii) male ≥ 60 years, or (iv) female < 50 or male < 60 years with a prior BMD showing evidence of osteoporosis, defined as a T-score of ≤  − 2.5 at the lumbar spine or femoral neck. Exclusion criteria were (1) index fracture of small bones (skull, face, cervical spine, hand, metatarsus, phalange, patella) or due to high trauma (motor vehicle accident or as per the investigator’s assessment), or (2) fragility fracture occurring < 5 years prior to index fracture to minimize the influence of a pre-index fracture on examined outcomes (Fig. 2).

### Study size

We planned to include approximately 750 patient charts from ≤ 120 primary care centers. We estimated each PCP would have 2–3 patients experiencing a fragility fracture annually, hence each could include 6–9. This study was planned to have descriptive endpoints.

### Variables of interest and outcome measures

Chart reviews began in January 2018. Thus, depending on when the index fracture occurred, the opportunity for follow-up was 1–4 years (2016–2017 to 2014–2017). For each patient, primary care sites recorded all fragility fractures starting from the date of index fracture until the date of chart review completion. We collected all prescribed osteoporosis treatments recorded in a patient’s chart within 1 year prior to the index fracture and for the duration of follow-up post-index fracture: bisphosphonates, denosumab, teriparatide, menopausal hormone therapy, calcitonin, and selective estrogen receptor modulators. Romosozumab was excluded as it was approved in Canada in 2019. Calcium and vitamin D intakes/supplements were not collected due to inconsistent documentation in patient records. Fracture risk assessment using the FRAX and/or CAROC tools, as well as dual-energy X-ray absorptiometry (DXA) BMD assessment (any skeletal site), were recorded beginning 5 years prior to the index fracture and for the duration of follow-up post-index fracture. The FRAX and CAROC tools were included in this study, consistent with the 2010 Canadian osteoporosis guidelines applicable to this cohort [21]. We described the proportion of patients with any fracture risk assessment (FRAX and/or CAROC) and with each type of fracture risk assessment (FRAX only, CAROC only, and both FRAX and CAROC), as well as the proportion of patients with BMD assessment. Among patients assessed for fracture risk, those with ≤  − 2.5 BMD T-score (lumbar spine, femoral neck, total hip, or other site) or high fracture risk (≥ 20% FRAX/CAROC probability for major osteoporotic fracture or ≥ 3% FRAX probability for hip fracture) were also recorded. Finally, osteoporosis diagnosis was collected ≤ 1 year prior to the index fracture and for the duration of follow-up post-index fracture, and defined based on the question posed to participating investigators, “Was the patient diagnosed with osteoporosis?” with the date of diagnosis indicated. The study cohort was described in terms of demographics, and co-morbid conditions relevant to osteoporosis care and glucocorticoid use (≥ 7.5 mg daily, ≥ 3 months) 1 year prior to index fracture until the end of follow-up.

### Data synthesis and analysis

The STROBE and RECORD statements were used to guide this report [25, 26]. Index fractures were described by skeletal sites and subsequent fractures by number, as outlined in Table 1. Median time from index to first subsequent fracture was calculated. Proportions of patients with osteoporosis treatment, fracture risk assessment (FRAX or CAROC), BMD assessment, or osteoporosis diagnosis documented at different time points relative to index fracture were described.

**Table 1:** Demographics and fracture details of the index fracture cohort

Characteristic	N=778% (n)
Females	80.5% (626)
Age^a^	
Mean ± SD	72.2 ± 10.9
Median (IQR)	73 (64-80)
Index fracture by site^b^	
Spine (clinical)	21.5% (167)
Radius	13.5% (105)
Hip	10.9% (85)
Ribs	9.8% (76)
Wrist	9.8% (76)
Humerus	8.5% (66)
Tarsals	6.8% (53)
Fibula	6.7% (52)
Tibia	4.1% (32)
Pelvis	3.6% (28)
Femur	3.3% (26)
Clavicle	2.3% (18)
Ulna	1.8% (14)
Knee	0.3% (2)
Scapula	0.3% (2)
Sternum	0.3% (2)
Number of subsequent fractures	
At least one	11.1% (86)
One	8.7% (68)
Two	1.9% (15)
Three	0.4% (3)

Values reported as % (n) unless otherwise indicated

*IQR* interquartile range, *SD* standard deviation

^a^*n*=776

^b^Percent of 778 index fracture cases

Four types of osteoporosis care gaps related to (1) treatment, (2) fracture risk assessment, (3) BMD assessment, and (4) osteoporosis diagnosis were characterized post-index fracture based on the 2010 Canadian osteoporosis guidelines applicable to this cohort [21]. To characterize the osteoporosis treatment gap, we reported on patients remaining untreated after their index fracture as well as after their subsequent fracture. However, notably, the 2010 guidelines only recommended treatment initiation following a hip, vertebral, or > 1 fragility fracture independent of other risk factors [21]. The fracture risk and BMD assessment gaps were characterized by reporting the proportion of patients without an assessment performed post-index fracture. The fracture risk assessment gap was further characterized by describing the number of patients with low versus moderate versus high fracture risk in those who had CAROC assessment performed both before and after index fracture. This was done to observe whether their index fracture was correctly incorporated into the CAROC risk calculations [21]. Finally, to characterize the gap, osteoporosis diagnosis rates were reported in relation to the index fracture date (before, at, after, or never), although the 2010 guidelines did not specify osteoporosis can be diagnosed solely based on a fragility fracture [21].

## Results

### Patients

Clinical and demographic characteristics of the index fracture cohort are listed in Table 1 and Online Resource 1. From 76 primary care centers, 778 patients (80.5% female) with a mean age of 72.2 years (range 44–105 years) were included in the full cohort. The most common index fractures occurred at the spine (21.5%, *n* = 167), radius (13.5%, *n* = 105), and hip (10.9%, *n* = 85). During the median (IQR) study follow-up of 363 (91–808) days, 11.1% (*n* = 86) of patients experienced ≥ 1 subsequent fracture, and of these, 41.9% (*n* = 36; 4.6% of 778) experienced it ≤ 1 year post-index fracture. No patients had glucocorticoids reported ≤ 1 year prior to index fracture. Co-morbid conditions relevant to osteoporosis care ranged between 0.3% (*n* = 2; stroke) and 19.3% (*n* = 150; type 2 diabetes). The available BMD and fracture risk assessment results are listed in Online Resource 2.

### Osteoporosis treatment patterns

**Fig. 3:**
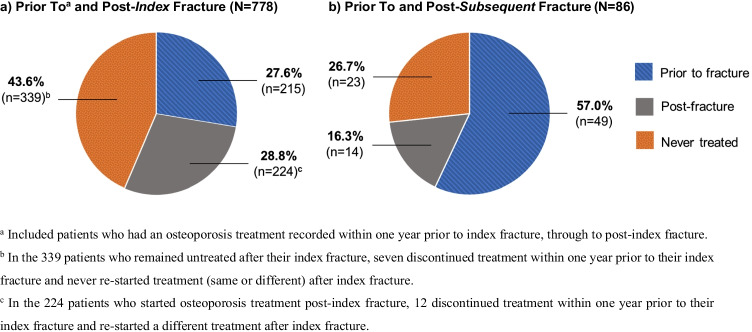
Osteoporosis treatment initiation

Of all 778 patients, 28.8% (*n* = 224) were started on osteoporosis therapy after their index fracture, and 27.6% (*n* = 215) were maintained on the same treatment initiated prior to their index fracture (Fig. 3a). When examining the 563 patients (72.4% of 778) not on osteoporosis therapy at the time of their index fracture, 39.8% (*n* = 224 of 563) were started on therapy post-index fracture, leaving the remaining 60.2% (*n* = 339 of 563) untreated until the end of the study follow-up (Fig. 3a). Of the patients with ≥ 1 subsequent fracture (*n* = 86; 11.1% of 778), 43.0% (*n* = 37 of 86) were untreated at the time of their subsequent fracture, and of these, 62.2% (*n* = 23 of 37) remained untreated thereafter (Fig. 3b). The most common osteoporosis treatment prior to or post-index fracture was bisphosphonate (70.7% prior; 61.6% post), followed by denosumab (22.7% prior; 47.8% post).

### Fracture risk assessment patterns

**Fig. 4:**
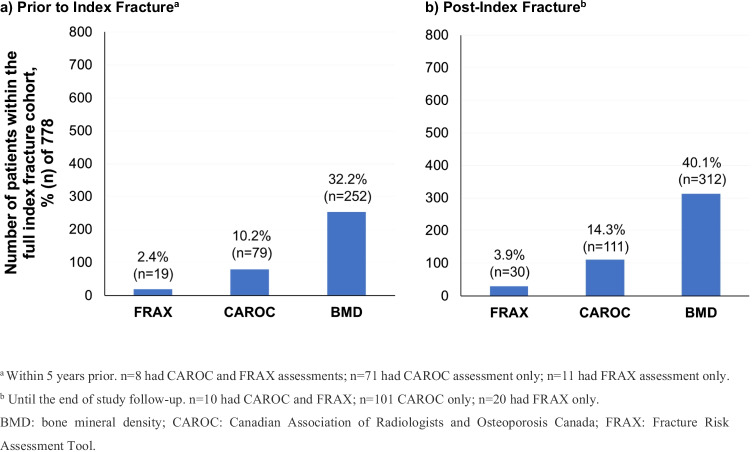
Fracture risk and BMD assessments

Of the full cohort, 11.6% (*n* = 90 of 778) had a FRAX and/or CAROC assessment completed ≤ 5 years prior to their index fracture (Fig. 4a) and 16.8% (*n* = 131 of 778) had a fracture risk assessment completed after their index fracture (Fig. 4b). The majority of post-fracture fracture risk assessments (61.7%; *n* = 87 of 141 assessments completed in 131 patients) were completed ≤ 6 months after the index fracture. The CAROC tool was used in more than 80% of patients with a fracture risk assessment before (87.8%, *n* = 79 of 90; Fig. 4a) or after (84.7%, *n* = 111 of 131; Fig. 4b). Of all 778 patients, only 43 (5.5%) patients had a FRAX and/or CAROC assessment completed both before and after their index fracture. A CAROC assessment was recorded both prior to and post-index fracture in 41 (5.3%) of all 778 patients. For CAROC assessments completed prior to the index fracture, 14 of the 41 patients (34.1%) were reported as low fracture risk, 20 of the 41 (48.8%) as moderate fracture risk and 7 of the 41 (17.1%) as high fracture risk (Online Resource 2). Conversely, post-index fracture, these numbers shifted to 8 (19.5%) as low risk, 13 (31.7%) as moderate risk, and 20 (48.8%) as high risk (Online Resource 2). Thus, in at least 36.6% of these patients (*n* = 15 of 41), the index fracture was not incorporated into the CAROC risk calculation, where a fragility fracture increases the risk category by one (i.e., from low to medium or medium to high risk) after initial estimation based on age and BMD alone. Eight patients were incorrectly estimated to be at low fracture risk post-fracture and at least seven patients were missing in the high-risk category.

### BMD assessment patterns

Of all 778 patients, 32.4% (*n* = 252) had a BMD assessment ≤ 5 years prior to their index fracture (Fig. 4a; Online Resource 2) and 40.1% (*n* = 312) after their index fracture (Fig. 4b; Online Resource 2) over the study follow-up. Of the latter patients, approximately half (52.9%, *n* = 165 of 312) had their BMD assessment completed ≤ 6 months after their index fracture.

### Osteoporosis diagnosis

**Fig. 5:**
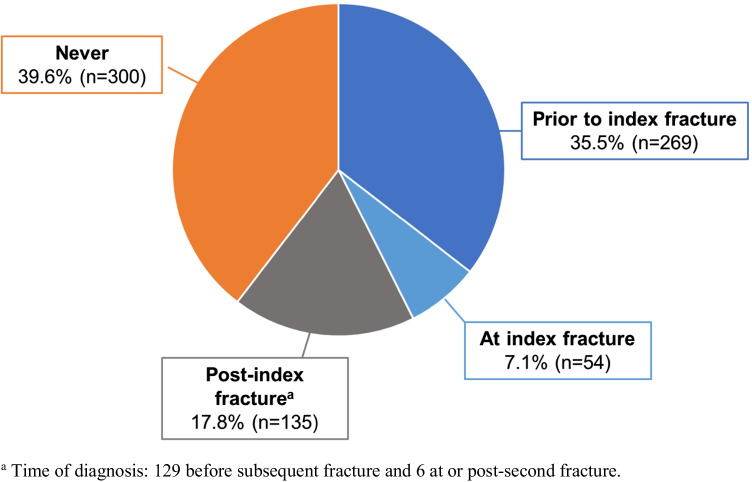
Osteoporosis diagnosis (*n* = 758)

The date of osteoporosis diagnosis was missing in 20 patients who were excluded from further assessment of pre-/post-index fracture diagnostic patterns due to the uncertainty of when the diagnosis occurred. Hence, of the remaining 758 patients, diagnosis was recorded in 35.5% (*n* = 269 of 758) prior to their index fracture (Fig. 5). In the remaining 489 patients undiagnosed prior to their index fracture, 38.7% (*n* = 189 of 489) had osteoporosis diagnosis recorded on the date of or after their index fracture, while 61.3% (*n* = 300 of 489) remained undiagnosed until the end of study follow-up (Fig. 5).

## Discussion

This study observed that a fragility fracture did not prompt osteoporosis diagnosis or treatment in 6 out of 10 (61.3% previously undiagnosed and 60.2% previously untreated) patients managed post-fracture in primary care. These data are noteworthy in the context of recent Endocrine Society, American Association of Clinical Endocrinologists, and International Osteoporosis Foundation guidelines for post-menopausal osteoporosis [1, 22, 24], which recommend a clinical diagnosis of osteoporosis and urgent initiation of pharmacotherapy following a fragility fracture at any osteoporotic fracture site, regardless of other risk factors. Although the 2010 Canadian guidelines (current during the surveillance period of this study) recommended treatment initiation only after hip, vertebral, or > 1 fragility fractures [21], substantial gaps in the uptake of the 2010 guidelines were still observed as 62.2% of patients with a subsequent fracture remained untreated thereafter. Further, a fragility fracture did not prompt fracture risk assessment in 83.2% of patients and did not prompt BMD assessment in 59.8% of patients, despite these recommendations in the applicable guidelines [21]. Taken together, these results signify that in the primary care setting, a fragility fracture is often not well recognized and/or acted upon as a serious clinical event requiring initiation of therapy to prevent subsequent fracture.

Compared to prior data, the current study suggests treatment rates post-fragility fracture are improving. Approximately twice as many patients received osteoporosis treatment (39.8%) compared to the < 20% rate reported by the Canadian Chronic Disease Surveillance System (CCDSS) in adults aged ≥ 65 during 2015–2016 [5]. It should, however, be noted that the CCDSS surveillance data included all fractures, which may have led to a lower treatment rate [5]. Given the subjectivity and potential confusion in identifying which fractures are low-trauma, international osteoporosis experts have recently urged the inclusion of high-trauma fractures in the clinical assessment for underlying osteoporosis and in the evaluation of future fracture risk, as both fractures showed a similar relationship with BMD and future fracture risk in a study of Canadian adults (aged ≥ 40 years) receiving a DXA scan between 1996 and 2016 [27].

Considering the low rate of fracture risk assessment specifically after a fracture (16.8%) in the current study, the importance of such assessment post-fracture was perhaps not adequately clarified in the guidelines applicable to this cohort [21, 28]. Nonetheless, of the fracture risk assessments completed after index fracture, 61.7% were completed within 6 months, suggesting the majority of PCPs who do recognize the prognostic significance of fragility fracture act relatively promptly. In addition, some patients with risk assessments completed prior to their fracture may have already been identified as high risk and on treatment. However, among clinicians using the CAROC tool, there is evidence that it was not applied correctly. More than one third (36.6%) of patients assessed were deemed to be at low risk of fracture, which is not possible in those with a prior fracture where the risk should be increased by 1 strata. It is important to qualify that incorrect use of the CAROC tool may have occurred at the radiologist setting and not by the referring PCP. Some radiologists in Canada provide CAROC risk calculations along with the BMD report shared with the PCP, but often rely on only age and BMD, excluding fracture history or other modifiers [2]. Furthermore, only 38.7% of patients were diagnosed with osteoporosis on or after the date of their index fracture, which suggests limited clinical recognition by the primary care or orthopedic team of the diagnostic significance of fragility fracture. However, our data cannot discriminate what proportion of diagnoses may have rather relied on a subsequent densitometric diagnosis. Thus, the need to recognize fragility fracture as a serious diagnostic clinical event influencing future fracture risk likely extends beyond primary care.

In addition, the low rates of fracture risk assessment observed post-fracture may reflect diversion of PCP attention and resources from osteoporosis to competing issues. Qualitative studies have shown that PCPs perceive osteoporosis management to be of lesser priority than other chronic conditions, such as cardiovascular disease [29, 30], despite research showing comparable 1-year mortality associated with a fragility fracture and myocardial infarction [13]. Strikingly, in a recent Canadian cohort of > 100,000 patients aged > 65, 21.5% of women and 32.3% of men died within 1 year following a hip fracture and 9.4% of women and 14.4% of men following a non-hip fracture [13].

Recognizing fracture as an important prognostic event has been urged by recent calls to action by the Public Health Agency of Canada and various international advocacy organizations as a key strategy toward helping address the secondary fracture prevention gap [5, 7, 8, 31]. In a recent Canadian cohort of older patients (aged > 65), median time between index fracture at any site and subsequent fracture was < 2 years [23]. Imminent fracture risk was also evident in the current study wherein 11.1% of patients had ≥ 1 subsequent fracture within a relatively short study follow-up period (median 363 days, IQR 91–808 days).

This observational study provides an update on post-fragility fracture care patterns in the Canadian primary care setting. Patient data were collected from across Canada with differing osteoporosis care priorities and barriers [15]. Our data support the findings from a recently published Canadian study demonstrating that diagnosis and treatment rates of osteoporosis after a fragility fracture of the hip are alarmingly low in the absence of a formal multidisciplinary treatment program [32]. However, our study has some important limitations. Three quarters (74.9%) of patients (and 75.9% of study PCPs) were from primary care centers in Ontario, the province accounting for approximately one third of all fractures and 36.1% of PCPs in Canada (total 46,797 PCPs) [5, 33]. Furthermore, Ontario is only one of two provinces with government action plans focusing on osteoporosis, and has among the best access to osteoporosis treatment and Fracture Liaison Services relative to other provinces [15]. Together, these factors may limit the generalizability of our findings to the entire Canadian population. Nonetheless, Ontario is the most populous province in Canada, inhabited by over 14 million of Canada’s 37 million population [34], hence the inherent value in understanding the osteoporosis practice patterns in this province. There is also potential for study site selection bias as specific PCPs were invited to participate in this study and may have been particularly interested in osteoporosis care. Collectively, all of these factors may underestimate the osteoporosis post-fracture care gap in Canadian primary care described herein and should be considered for future research. Furthermore, the scope of this study was to observe fracture risk and BMD assessment in a real-world setting, and the participating centers were not asked to ascertain pre-/post-index fracture risk based on the available chart data, thus leaving only a small number of patients with available BMD and fracture risk results pre-/post-index fracture. Finally, whether osteoporosis diagnosis and treatment initiation were based on BMD or fracture remains unknown in the current study and should be documented in future studies.

## Conclusion

In this post-fragility fracture cohort, 6 in 10 patients did not receive osteoporosis treatment and 8 in 10 patients did not receive fracture risk assessment after their fragility fracture. Even after experiencing further fragility fractures over a relatively short follow-up, 6 in 10 patients remained untreated. This mismatch potentially signifies that, in the primary care setting, a fragility fracture is not well recognized as a sentinel clinical event. Our data underscore the pressing need to support PCPs in integrating current evidence-based secondary fracture prevention practices, with particular value in facilitating timely incorporation of fragility fracture events into their evaluation and management of patients with osteoporosis.

## Supplementary Information

Below is the link to the electronic supplementary material.

Supplementary file1 (DOCX 23 KB)
