# Supplementary material for: A retrospective observational study of osteoporosis management after a fragility fracture in primary care
Source: Arch Osteoporos. 2022 May 6;17(1):75. doi: 10.1007/s11657-022-01110-z (PMC9072526; doi:10.1007/s11657-022-01110-z)
Supplement: Supplementary file 1 — Supplementary file1 (DOCX 23 KB) [file 11657_2022_1110_MOESM1_ESM.docx]

**Supplementary Information**

**Archives of Osteoporosis**

**Title:** A Retrospective Observational Study of Osteoporosis Management after a Fragility Fracture in Primary Care

**Authors:** Alan Bell^1^, David L. Kendler^2^, Aliya A. Khan^3^, Marla Shapiro^4^, Anne Morisset^5^, Jean-Pierre Leung^6^, Maureen Reiner^7^, Stephen M. Colgan^7^, Lubomira Slatkovska^8^, Millicent Packalen^8^

**Affiliations:**

^1^Department of Family and Community Medicine, University of Toronto, Toronto, ON, Canada

^2^Department of Medicine, Division of Endocrinology, University of British Columbia, Vancouver, BC, Canada

^3^Department of Medicine, Divisions of Endocrinology and Metabolism and Geriatrics, McMaster University, Hamilton, ON, Canada

^4^Department of Family and Community Medicine, University of Toronto, Toronto, ON, Canada

^5^Department of Medicine, Division of Internal Medicine, Sherbrooke University, Sherbrooke, QC, Canada

^6^Department of Family Medicine, University of Calgary, Calgary, AB, Canada

^7^Amgen Inc., California, USA

^8^Amgen Canada Inc., Mississauga, ON, Canada

**Corresponding author:** Millicent Packalen: mhume@amgen.com

**Supplementary Information:**

**Online Resource 1.** Clinical and Demographic Characteristics of the Index Fracture Cohort

**Online Resource 2.** BMD and Fracture Risk Assessment Results in the Full Index Fracture Cohort and Patients on Osteoporosis Treatment at Their Index Fracture Date

**Online Resource 1. Clinical and Demographic Characteristics of the Index Fracture Cohort**

| **Characteristic** | **% (n)** |
| --- | --- |
| **Total number of patients** | 778 |
| **Sex**  Female  Male | 80.5% (626)  19.5% (152) |
| **Age^a^**  Mean ± SD  Median (IQR) | 72.2 ± 10.9  73 (64-80) |
| **Province**  Alberta  British Columbia  Manitoba  New Brunswick  Ontario Quebec | 2.6% (20)  13.5% (105)  0.4% (3)  1.9% (15)  74.9% (583)  6.7% (52) |
| **Respiratory conditions^b^**  Asthma  COPD | 6.7% (52)  8.4% (65) |
| **Inflammatory conditions^b^**  Psoriasis  Rheumatoid arthritis  Spondyloarthritis | 1.9% (15)  3.0% (23)  3.9% (30) |
| **Cancer^b^** | 4.9% (38) |
| **Chronic kidney disease^b^** | 7.6% (59) |
| **Diabetes^b^**  Type 1 diabetes  Type 2 diabetes | 0.5% (4)  19.3% (150) |
| **Vascular events^b^**  Myocardial infarction  Stroke | 0.4% (3)  0.3% (2) |
| **Dementia^b^** | 3.3% (26) |
| **Index fracture by site^c^**  Spine (clinical)  Radius  Hip  Ribs  Wrist  Humerus  Tarsals  Fibula  Tibia  Pelvis  Femur  Clavicle  Ulna  Knee  Scapula  Sternum | 21.5% (167)  13.5% (105)  10.9% (85)  9.8% (76)  9.8% (76)  8.5% (66)  6.8% (53)  6.7% (52)  4.1% (32)  3.6% (28)  3.3% (26)  2.3% (18)  1.8% (14)  0.3% (2)  0.3% (2)  0.3% (2) |
| **Number of subsequent fractures**  At least one  One  Two  Three | 11.1% (86)  8.7% (68)  1.9% (15)  0.4% (3) |

Values reported as % (n) unless otherwise indicated.

^a^ n=776.

^b^ Time frame for comorbidities was any time prior to the index fracture date.

^c^ Percent of total number of index fracture cases (N=778) in select primary care centres across Canada from January 1, 2014 to December 1, 2016. Reported from highest to lowest number.

COPD: chronic obstructive pulmonary disease; IQR: interquartile range; SD: standard deviation.

**Online Resource 2. BMD and Fracture Risk Assessment Results in the Full Index Fracture Cohort and Patients on Osteoporosis Treatment at Their Index Fracture Date**

| **Assessment** | **Full cohort (N=778)** | **Patients on Osteoporosis Treatment at Index Date (N=215)** |
| --- | --- | --- |
|  | **% (n)^a^** | **% (n)^a^** |
| **T score ≤-2.5**  Prior to index  Post-index | 32.1% (45/140)  29.1% (55/189) | 51.5% (34/66)  46.2% (24/52) |
| **FRAX: high risk of fracture^b^**  Prior to index  Post-index | 50.0% (9/18)  30.0% (9/30) | 50.0% (7/14)  50.0% (2/4) |
| **FRAX: high risk of hip fracture^b^**  Prior to index  Post-index | 78.6% (11/14)  42.3% (11/26) | 88.9% (8/9)  50.0% (1/2) |
| **CAROC: high risk of fracture^b^**  Prior to index  Post-index | 30.4% (24/79)  50.0% (53/106) | 59.0% (23/39)  64.7% (22/34) |

^a^ Data represents patients who had results available. Patients assessed prior to vs. post-index fracture were not necessarily the same patients.

^b^ Defined as FRAX/CAROC ≥20% probability for major osteoporotic fracture or FRAX ≥3% probability for hip fracture.

BMD: bone mineral density; CAROC: Canadian Association of Radiologists and Osteoporosis Canada; FRAX: Fracture Risk Assessment Tool.
